# Transcriptional Instability during Evolving Sepsis May Limit Biomarker Based Risk Stratification

**DOI:** 10.1371/journal.pone.0060501

**Published:** 2013-03-27

**Authors:** Antonia Kwan, Mike Hubank, Asrar Rashid, Nigel Klein, Mark J. Peters

**Affiliations:** 1 Infectious Diseases and Microbiology Unit, Institute of Child Health, University College London, London, United Kingdom; 2 Department of Pediatrics, University of California San Francisco, San Francisco, California, United States of America; 3 Molecular Haematology & Cancer Biology Unit, Institute of Child Health, University College London, London, United Kingdom; 4 Queens Medical Centre, Nottingham University Hospitals NHS Trust, Nottingham, United Kingdom; 5 Portex Unit for Paediatric Critical Care, Institute of Child Health, University College London, London, United Kingdom; Rutgers University, United States of America

## Abstract

**Background:**

Sepsis causes extensive morbidity and mortality in children worldwide. Prompt recognition and timely treatment of sepsis is critical in reducing morbidity and mortality. Genomic approaches are used to discover novel pathways, therapeutic targets and biomarkers. These may facilitate diagnosis and risk stratification to tailor treatment strategies.

**Objective:**

To investigate the temporal gene expression during the evolution of sepsis induced multi-organ failure in response to a single organism, *Neisseria meningitidis*, in previously healthy children.

**Method:**

RNA was extracted from serial blood samples (6 time points over 48 hours from presentation) from five critically ill children with meningococcal sepsis. Extracted RNA was hybridized to Affymetrix arrays. The RNA underwent strict quality control and standardized quantitation. Gene expression results were analyzed using GeneSpring software and Ingenuity Pathway Analysis.

**Result:**

A marked variability in differential gene expression was observed between time points and between patients revealing dynamic expression changes during the evolution of sepsis. While there was evidence of time-dependent changes in expected gene networks including those involving immune responses and inflammatory pathways, temporal variation was also evident in specific “biomarkers” that have been proposed for diagnostic and risk stratification functions. The extent and nature of this variability was not readily explained by clinical phenotype.

**Conclusion:**

This is the first study of its kind detailing extensive expression changes in children during the evolution of sepsis. This highlights a limitation of static or single time point biomarker estimation. Serial estimations or more comprehensive network approaches may be required to optimize risk stratification in complex, time-critical conditions such as evolving sepsis.

## Introduction

Sepsis-induced multiple organ failure and the related systemic inflammatory response syndrome contribute to the vast majority of deaths during critical illness in adults and children [1]. New therapies for sepsis-induced multiple organ failure are as far as ever from delivering survival benefits [2]. Interventional studies are limited by the heterogeneity of these clinically defined syndromes of sepsis [3], which also undermine specific anti-inflammatory therapies [4].

In children, pediatric septic shock continues to be an important health problem, and despite the development of effective antibiotics, vaccines and intensive care unit-based support, a major challenge remains the early recognition of septic shock, which has been shown to be linked to improved survival [5]. Early recognition and timely management of meningococcal septicemia has reduced mortality rate from 20–40% in the late 90s to 5–20% over the last decade [6]. Clinical predictors of poor outcome have been recognized for many years and include a low core body temperature, seizures or shock on initial presentation, neutropenia, thrombocytopenia, and purpura fulminans [7]. Despite detailed knowledge of the immunopathology [8] and new developments in genomics and proteomics, this has yet to be translated into clinically useful risk prediction.

Several groups have used genomic approaches to probe further the global gene expression changes during sepsis [9], [10]. Biomarkers [11] or more realistically patterns on genome-wide expression arrays have the potential to provide vital diagnostic and risk stratification functions [10], [12], [13]. Gene expression studies using this approach have been limited to one or two time points providing snapshots of gene expression during the course of sepsis.

In this study, we investigated the influence of time on the expression of 28,725 genes in the earliest phases of development of sepsis-induced multiple organ failure (0, 4, 8, 12, 24 and 48 hours) in response to a single pathogen (*N. meningitis*) in a cohort of children with no pre-existing diseases. We observed an increasing number of genes that were differentially regulated from admission (surrogate baseline at 0 hours) with time over the first 48-hours, showing marked expression instability. This degree of transcript expression instability was also reflected in biomarker genes that have been suggested for risk stratification in sepsis [14]. As such, the changing transcript expression during sepsis reflects the dynamic nature of the disease evolution, and is therefore a challenge to microarray analysis for diagnosis and risk stratification.

## Materials and Methods

### Patient recruitment

This study was approved by the Nottingham University ethics committee (REC reference 05/Q2403/53). Patients presenting to Nottingham University Hospital Paediatric Intensive Care Unit (PICU) were recruited, and parents gave full written consent on behalf of children who took part in the study. The patients recruited received the standard clinical treatment, including appropriate antimicrobial therapy for presumed meningococcal meningitis. Four of the patients subsequently had the diagnosis of meningococcal Group B bacteria made following polymerase chain reaction.

### Blood sampling

Blood samples were collected from 5 patients on admission to PICU (designated 0 hours) and subsequently at 4, 8, 12, 24, and 48 hours after their admission. Blood was collected, mixed with PAXgene Blood RNA reagent and left to incubate at room temperature before freezing at −80°C.

### RNA extraction

Blood and PAXgene Blood RNA reagent were thawed, incubated at room temperature, and RNA was purified according to the Qiagen PAXgene Blood RNA manual protocol using the commercial kit (PAXgene Blood RNA kit, Qiagen). Further ethanol precipitation steps were necessary to achieve adequate RNA concentration and purity, which were assessed by spectrophotometry (A260/A280 ratio, NanoDrop ND-1000 spectrophotometer) and capillary electrophoresis (Agilent 2100 Bioanalyzer, Agilent Inc). The 4-hour sample from Patient 4 was degraded and was not used for further microarray experiments.

### cRNA synthesis and chip hybridization

cRNA synthesis was performed using Ambion^®^ WT Expression kit, starting with 250 ng total RNA. Following cRNA digestion with RNase H, labeled cDNA was hybridized onto Human Gene 1.0 ST Arrays (Affymetrix^®^), and processed according to the protocol outlined by Affymetrix.

### Microarray data analysis

A total of 33,297 probe sets from 29 arrays on the Human Gene 1.0 ST Arrays were analyzed. Complete gene expression data for each patient are available on the ArrayExpress database (EMBL-EBI ArrayExpress accession number E-MEXP-3850; http://www.ebi.ac.uk/arrayexpress/). The raw data files were imported to GeneSpringGX (version 11.5, Agilent Technologies), normalized and log-transformed according to the workflow pipeline. Control probe sets, as well as probes absent on all arrays were excluded from analysis. Probe sets that differed by ≥2 fold change between 0-hour samples across the 5 patients were also excluded from further analysis, resulting in 28,725 probe sets analyzed. For each patient, probe sets were analyzed for ≥2-fold change differences between subsequent time points compared to the respective 0-hour sample, resulting in 5 pairwise comparisons for each patient (apart from patient 4 which did not include a 4-hour sample due to RNA degradation).

### Identification of significant pathways

Gene lists generated from GeneSpring were formatted and uploaded to Ingenuity^®^ Pathway Analysis (IPA). This is a web-based analysis tool in which published scientific findings have been systematically encoded into an ontology and modeled into molecular networks based on published physical, transcriptional or enzymatic interactions. Fisher’s exact test was used to calculate a *p*-value determining the probability that each biological function assigned to that dataset is due to chance alone. Networks were further analyzed for relevance to the particular biological system and clinical setting.

## Results

We studied 5 previously healthy children with a clinical diagnosis of acute meningococcal septicemia with sepsis-induced multiple organ failure. Their clinical presentation patterns of organ failures were similar, as were antimicrobial therapy and approaches to (but not extent of) organ support (Table 1). Gene expression in whole blood was determined at time 0 hours (on admission to pediatric intensive care) and at 4, 8, 12, 24 and 48 hours into their intensive care admission; in total 30 blood samples were collected.

**Table 1:** Patient demographics for longitudinal analysis.

Patient	1	2	3	4	5
Number of samples	5	5	5	5*	5
Age (months)	13	10	22	24	9
Sex	Female	Female	Female	Male	Male
Duration of PICU admission (days)	9	4	3	6	3
No. of organ(s) in failure	4	4	3	3	3
PELOD score on admission	61	31	31	12	11
PELOD score at 24 hours	52	2	22	22	2
PELOD score at 48 hours	43	2	31	12	1
Median PRISM score	6	6	6	6	6
Infection	Negative culture (Presumed meningococcal sepsis)	GpB meningococcus	GpB meningococcus	GpB meningococcus	GpB meningococcus
Mortality (at 28 days)	Died	Alive	Alive	Alive	Alive

PELOD, PEdiatric Logistic Organ Dysfunction Score; PRISM, Pediatric Risk of Mortality Score.

* 4-hour sample resulted in degraded RNA, not used for further array experiments.

**Figure 1:**
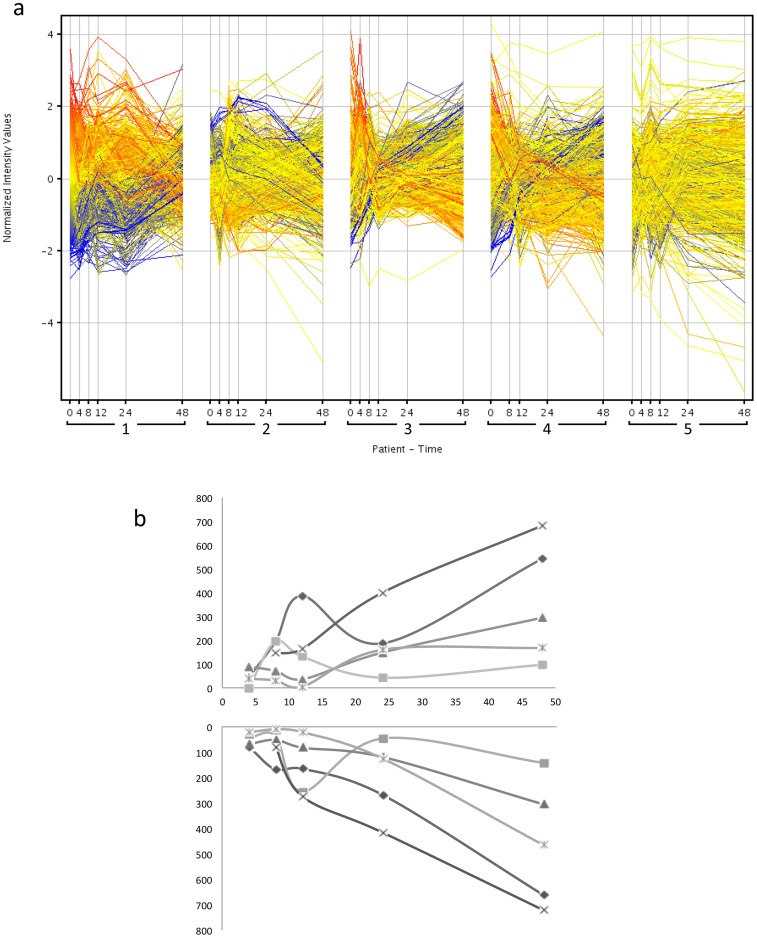
Gene expression changes over time. (a) Plot of 28,869 genes with normalized intensity values against time for the 5 patients, from GeneSpring 11.5 analysis (see Methods). Colors of plots are representative of genes' change in expression level compared to the corresponding gene's expression at 0 hours for patient 1. Blue is for upregulated expression, yellow is for no change in expression, and red is for downregulated expression compared to patient 1 at time 0. (b) Numbers of genes up- and down-regulated over time for each of the 5 patients, compared to each patient's gene expression at 0 hours. The top panel indicates numbers of up-regulated genes, the bottom panel indicates numbers of down-regulated genes, for patients 1 (•), 2 (▪), 3 (Δ), 4 (**x**), and 5 (*).

Twenty-nine microarrays were hybridized, as RNA from the 4-hour sample from patient 4 had degraded and was not used for further array experiments. GeneSpring GX software (Agilent Technologies) was used for initial visualization (Figure 1a) and subsequent analysis of the microarray data. Figure 1a demonstrates the complex temporal relationship of disease evolution and instability of transcript expression that occur with time. In order to increase the comparability between patients in subsequent analysis, the signal intensities of 28,869 gene probes of the 0-hour samples were compared between all 5 patients; 144 gene probes (0.05% of total number of gene probes) were found to vary across the patients by ≥2 fold. Such baseline differences were interpreted as differential gene expression downstream to events prior to PICU admission. To aid comparisons between patients, the differentially regulated genes at baseline were excluded from subsequent analysis. Further analysis of the remaining 28,725 gene probes showed differential up- and down- regulation of genes during the time-course, compared to baseline at admission (0 hours), for all 5 patients, with the general trend of increasing numbers of up- and down-regulated genes with time, seen in varying degrees (Figure 1b).

**Figure 2:**
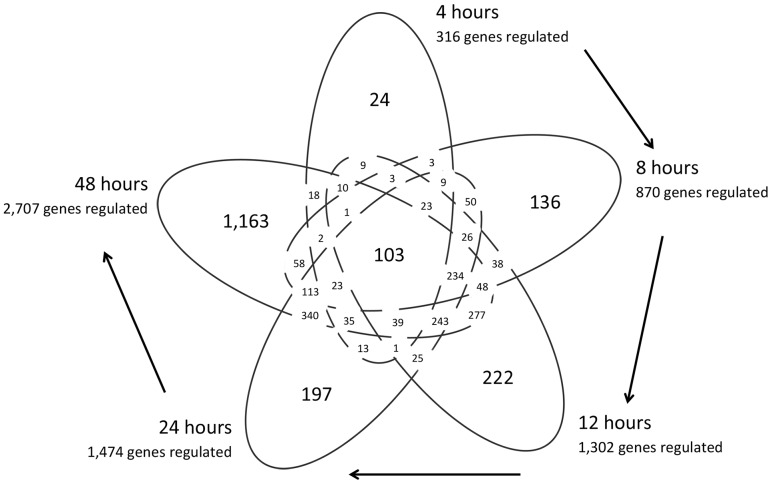
Temporal variation in gene expression changes. Numbers of genes that change at different time points as compared to their baseline gene expression level are shown. Expression levels were analyzed by pairwise comparisons between each of 5 time points (4, 8, 12, 24, and 48 hours) compared to each patient's corresponding 0 hour gene expression profiles, and expression levels of greater than 2-fold changes were noted. This was performed for all 5 patients (except in patient 4 where the 4-hour sample had degraded RNA), 24 pairwise comparisons in total. Numbers in the sections of the Venn diagram correspond to numbers of genes that were up- or down-regulated by ≥2-fold for at least 1 in the 5 patients; numbers in the outer sections correspond to the numbers of genes uniquely regulated at the corresponding time points, and the number in the middle corresponds to the number of genes differentially regulated at all time points during the time-course.

To determine differential gene expression at specific time points, genes were grouped based on differential regulation compared to baseline at admission (≥2 fold change in gene expression compared to gene expression levels at 0 hours) at the 5 subsequently sampled time points (4, 8, 12, 24, 48 hours). Genes were found to be differentially up- or down-regulated at all time points throughout the time-course compared to baseline, or were up- or down-regulated only at distinct time points, with others overlapping in expression at different time points (Figure 2). The complexity in gene regulation is clearly demonstrated in this clinical setting; gene expression changes continue to occur in patients with evolving meningococcal sepsis beyond 24 and 48 hours.

**Figure 3:**
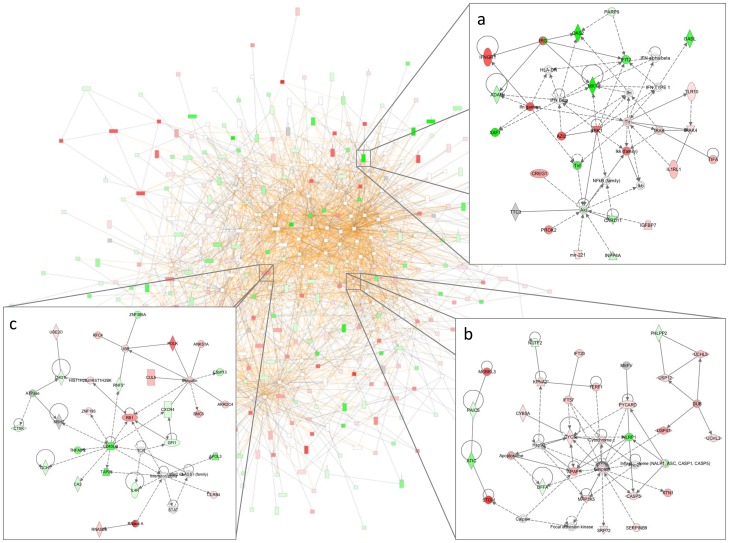
Ingenuity Pathway Analysis network. Example of a gene network derived from genes uniquely regulated at 48 hours, comprising 3 merged networks consisting of 444 genes (red, increased expression; green, decreased expression) as derived from Ingenuity Pathway Analysis. Selected regions of the network are highlighted, consisting of genes involved in: (a) Antimicrobial response, Inflammatory response, Cell-To-Cell Signaling and Interaction (“*MX1* genes”, network score 23); (b) Cell Death, Cell Signaling, DNA Replication, Recombination and Repair (“*CASP* genes”, network score 33); (c) Cellular Development, Hematological System Development and Function, Hematopoiesis (“*RB1* genes”, network score 37).

Networks were generated in IPA using differentially regulated genes (≥2-fold change) between downstream time points compared to baseline at admission to elucidate biological networks activated during the various time points in the evolution of sepsis. The mapping of genes to biological networks was accompanied by a network score, which describes the likelihood that a particular group of genes are found in the network by chance. The higher the number of regulated genes within the network, the higher the network score, which relates to the negative exponent of the respective *p*-value (i.e. network score = −log_10_(*p*-value)). Genes regulated at 4 hours included *CXCR5*, *HLA-DQB1*, *HLA-DOA*, and were associated with the “antigen presentation, cellular compromise, infectious disease, respiratory disease” network (network score 46). Genes regulated at 8 hours included *TNF*, *CD86*, *IL27RA*, *MT1F*, and were associated with “cell-to-cell signaling, inflammatory response, immune cell trafficking” networks (network scores 25–33). Genes regulated at 12 hours included *HLA-DRB3*, *HLA-DPB1*, *ZNF32*, *ZBTB16* and were associated with networks relating to “cellular growth and proliferation, cellular assembly and organization, cell-mediated immune responses, cell death” (network scores 28–44). At 24 hours, genes regulated were associated with similar networks highlighted at 12 hours, with the addition of networks relating to “cell cycle, cell morphology, cellular compromise, DNA replication, recombination and repair, drug metabolism” (network scores 30–58). This group of genes included *CASP1*, *SAMHD1*, *ZC3H11A*, *CDC26*. By 48 hours, differentially regulated genes were associated with similar networks to those reported at 24 hours, but with the addition of “protein synthesis” (network scores 33–42). These genes include *XAF1*, *MX1*, *OAS2*, *CD28*, *BAZ1A*. The annotated gene networks populated by genes found to be uniquely regulated at different time points in this group of patients are consistent with the temporal evolution of the biological response to meningococcal sepsis (Figure 3).

**Figure 4:**
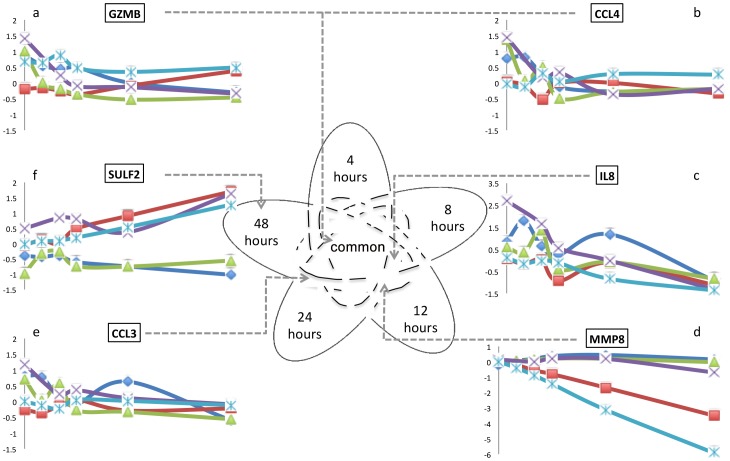
Expression variability of proposed biomarkers for sepsis. The gene expression changes of a selection of proposed biomarkers are shown, to demonstrate their variability over the time-course. Starting from (a) in clockwise direction: (a) GZMB and (b) CCL4 are up- or down-regulated compared to 0 hours at all time points (“common”); (c) IL8 is up- or down-regulated compared to 0 hours at 8, 12, 24, and 48 hours; (d) MMP8 is up- or down-regulated compared to 0 hours at 12, 24, and 48 hours; (e) CCL3 is up- or down-regulated compared to 0 hours at 24 and 48 hours; (f) SULF2 is up- or down-regulated compared to 0 hours at 48 hours only. Up- and down-regulation is filtered at ≥2-fold change compared to 0 hours.

Stratification strategies have been suggested that utilize single or, more recently, multiple biomarkers as predictors of outcome in sepsis, taking into account the complexity and heterogeneity of this clinical syndrome [14]. In this study, the transcript levels of the panel of 15 suggested biomarkers varied differentially with time: from *ELA2* that did not change during the sampled time-course, to *HSPA1B*, *CCL4*, *GZMB* and *ORM1* that varied across the whole time-course (Table 2). A selection of the 15 suggested biomarkers are shown in Figure 4 as transcript expression level over time in the 5 patients included in this study, and demonstrates the differential changes in transcript levels across the different biomarkers.

**Table 2:** Biomarkers and their change in expression over time-course.

Gene	No change from baseline	Change at 4, 8, 12, 24, 48 hours	Change at 8, 12, 24, 48 hours	Change at 12, 24, 48 hours	Change at 24, 48 hours	Change at 48 hours
*ELA2*	*					
*HSPA1B*		*				
*CCL4*		*				
*GZMB*		*				
*ORM1*		*				
*IL1A*			*			
*LTF*			*			
*IL8*			*			
*RETN*			*			
*THBS1*			*			
*MMP8*				*		
*LCN2*				*		
*FGL2*				*		
*CCL3*					*	
*SULF2*						*

The 15 suggested biomarkers varied in their expression in the 5 patients with meningococcal sepsis, ranging from no change in expression as compared to baseline at 0 hours, to changes at 4, 8, 12, 24, and 48 hours as compared to baseline. This demonstrates a temporal grouping of genes, which may facilitate placing patients in their evolving clinical course of sepsis.

## Discussion and Conclusion

This study demonstrates the extent of the complexity of temporal changes in gene expression that occur during the evolution of sepsis-induced multiple organ failure. The frequent sampling of individual cases provide a novel insight into the rate of change of expression with time.

Longitudinal genomic expression profiling analyses in pediatric septic shock has been reported previously at a lower sampling frequency (days 1 and 3) [13]. Our study provides a high resolution temporal analysis of gene expression changes in the earliest stages of evolving pediatric sepsis. The extent and persistence of the variability we observed contrasted strikingly with clinical work and Calvano *et al*'s volunteer endotoxin model of systemic inflammation where perturbations in gene expression returned to baseline by 24 hours [15].

We undertook this study because the majority of deaths from sepsis in children occur in the first 6 to 24 hours [3], [16], [17]. Therefore any proposed risk stratification tool must be available promptly. If not, it will be limited to describing outcomes in the subset of cases that have survived the initial hazardous period.

Our study has advantages including the high sampling frequency but also the focus on cases with an acute presentation of previously healthy children infected with a single pathogen. These features would be expected to remove several sources of variability. Despite these common features, the variability in expression is striking. There was wide variability in expression of existing candidate biomarkers especially within the first 24 hours. This variability in transcript level expression displayed by the panel of 15 candidate genes suggest their utility as a protein biomarker may be limited. It may be that change in expression levels of biomarkers are of as much significance as absolute levels.

One of the suggested biomarkers, matrix metalloproteinase 8 (MMP8), has attracted interest as a marker of sepsis severity [18]. Mining previous microarray data, MMP8 transcript expression was increased in children with septic shock, with higher levels in non-survivors [18]. This suggests that MMP8 can be used as a marker of disease severity. Interestingly, in our patients, the MMP8 expression varied with time across the 5 patients (Figure 4d). While MMP8 levels were very similar on admission to intensive care, there was clear divergence by 12 hours, which persisted at 24 and 48 hours. The degree of organ dysfunction was markedly greater in patients 1, 3, 4, and this is reflected in the higher MMP8 expression levels compared to patients 2 and 5. However, the differences in MMP8 expression levels were not detected on admission to intensive care; rather, the divergence in MMP8 transcript expression was only reliably detected at 12 hours into their admission. This supports the need for temporal consideration in using biomarkers for risk stratification; a single “snapshot” may be less informative than a trend.

The main weakness of this work is our tight focus on 5 children. Comparisons with more children with sepsis from other organisms and with systemic inflammation from non-septic causes are essential future steps. In addition, correlations between gene expression changes and protein levels for biomarker assessment are required. Finally and most challenging would be generating sufficient power to match specific gene (or gene network) expression patterns with specific clinical phenotypes. A further caveat may be the limitation of using time 0 on admission as a reference baseline. There are wide differences in the pre-hospital course influencing time of presentation to medical attention and admission to intensive care. This may have an impact on the observed biologic variability. A potential solution may be to standardize to peak expression levels of a selected biomarker gene, but the problem is that this can only be identified retrospectively. Other solutions include attempting to take into account lead-time between initial symptoms and presentation to medical services into the analysis. The biologic impact of this variability in timings warrants further exploration.

This study shows that while transcriptional profiling is very powerful for gaining insight into the interplay between molecular networks, temporal analyses are required to fully exploit the full potential of this approach for both understanding disease evolution and detecting biomarkers in an evolving clinical syndrome such as sepsis.
